# A Novel Pathway for a Tumor Suppressor

**DOI:** 10.1371/journal.pbio.0020339

**Published:** 2004-09-07

Millions of different proteins exist in nature, each with a unique structure that determines its function. Proteins can have different effects depending on where they are in the cell and which proteins or pathways they associate with. The number of proteins produced by a cell varies, but scientists estimate that the human genome produces some 100,000 proteins, and with thousands of proteins likely to be active in a single cell, it's inevitable that the molecular components of cellular pathways overlap. It is thought that this may be the case for a tumor suppressor named VHL (after its role in von Hippel-Lindau disease, an inherited cancer syndrome that predisposes affected individuals to kidney and vascular tumors).[Fig pbio-0020339-g001]

**Figure pbio-0020339-g001:**
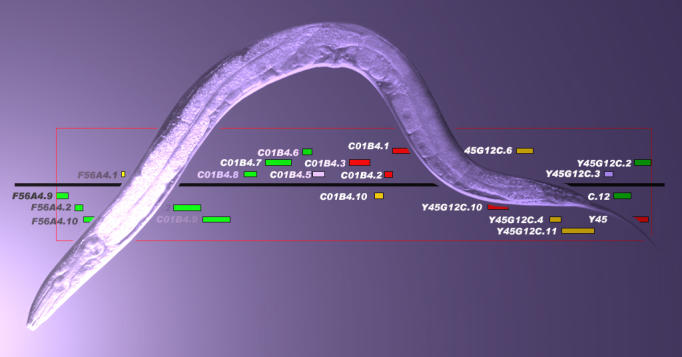
Genetic evidence for HIF-independent VHL-regulated pathways

The broad strokes of VHL action have been outlined: VHL is a ubiquitin ligase, an enzyme that targets proteins for destruction. VHL's best characterized target is a transcription factor called hypoxia-inducible factor 1 (HIF-1). When oxygen levels drop below normal—a condition called hypoxia—HIF-1 proteins are not degraded and may enter the nucleus, where they trigger the transcription of roughly 100 genes whose proteins either increase oxygen delivery or engage metabolic pathways that help the cell adapt to hypoxia. Scientists have long suspected that VHL has other targets, yet only HIF-1 has been clearly established. Now Peter Ratcliffe and colleagues use genetic methods in the nematode Caenorhabditis elegans to provide direct evidence of a HIF-1-independent function of VHL.

Previous studies have shown that when cells lack VHL proteins, HIF-1 is not degraded, resulting in the overexpression of HIF target genes. VHL-defective cells also show abnormalities in the extracellular matrix, the structural scaffolding that surrounds the cell. But it has not been clear whether these effects stem from HIF-1 dysregulation or something else.

To disentangle the actions of the two proteins, Ratcliffe and colleagues compared the consequences of VHL protein inactivation in a variety of genetic backgrounds in C. elegans. Then they analyzed the gene expression profiles of each of these mutant strains to identify pathways that required VHL but not HIF-1. To their surprise, the authors found, “all of the VHL-regulated genes fell into one of two patterns.” Their expression was either independent of HIF-1 and dependent on a range of genes associated with the extracellular matrix, or vice versa. What's more, these gene sets fell into distinct categories based on their chromosomal location, predicted functional similarities, and pattern of dysregulation.

These results, Ratcliffe and colleagues conclude, reflect the disruption of “two discrete aspects of VHL function.” One depends on HIF-1—inhibiting the transcription factor when oxygen concentrations are normal—and one doesn't; disruption of this HIF-1-independent function produces defects similar to those seen in mutants with defects in extracellular matrix assembly. These results also fall in line with other studies that have linked VHL deficiency to extracellular matrix defects, though the precise link remains unclear. It's also not clear whether this HIF-1-independent function means that VHL is still functioning as a ubiquitin ligase but targeting a different substrate or whether it represents a completely different function of VHL. For now, direct evidence of a HIF-1-independent pathway for VHL charts a clear path for researchers interested in pinning down the functions of this undoubtedly multidimensional ubiquitin ligase. It also gives VHL syndrome researchers—who have long suspected that other functions of the VHL tumor suppressor play a role in the onset of the disease—a promising lead to explore.

